# Molecular testing and analysis of disease spreading during the emergence of COVID-19 in Macaé, the Brazilian National Capital of Oil

**DOI:** 10.1038/s41598-021-99475-7

**Published:** 2021-10-11

**Authors:** Natália Martins Feitosa, Bruno da Costa Rodrigues, Ana Cristina Petry, Keity Jaqueline Chagas Vilela Nocchi, Rodrigo de Moraes Brindeiro, Carla Zilberberg, Cintia Monteiro-de-Barros, Flavia Borges Mury, Jackson de Souza-Menezes, José Luciano Nepomuceno-Silva, Manuela Leal da Silva, Marcio José de Medeiros, Raquel de Souza Gestinari, Alessandra da Silva de Alvarenga, Allan Pierre Bonetti Pozzobon, Carina Azevedo Oliveira Silva, Daniele das Graças dos Santos, Diego Henrique Silvestre, Graziele Fonseca de Sousa, Janimayri Forastieri de Almeida, Jhenifer Nascimento da Silva, Layza Mendes Brandão, Leandro de Oliveira Drummond, Lupis Ribeiro Gomes Neto, Raphael de Mello Carpes, Renata Coutinho dos Santos, Taynan Motta Portal, Amilcar Tanuri, Rodrigo Nunes-da-Fonseca

**Affiliations:** 1 Instituto de Biodiversidade e Sustentabilidade-NUPEM, Universidade Federal do Rio de Janeiro (UFRJ), Av. São José do Barreto 764, Macaé, 27965-550 Brazil; 2 Laboratório de Virologia Molecular, Departamento de Genética, Instituto de Biologia, Universidade Federal do Rio de Janeiro (UFRJ), Rio de Janeiro, 21941-902 Brazil

**Keywords:** Viral infection, PCR-based techniques, Evolution, Genetics, Molecular biology

## Abstract

The Brazilian strategy to overcome the spread of COVID-19 has been particularly criticized due to the lack of a national coordinating effort and an appropriate testing program. Here, a successful approach to control the spread of COVID-19 transmission is described by the engagement of public (university and governance) and private sectors (hospitals and oil companies) in Macaé, state of Rio de Janeiro, Brazil, a city known as the National Oil Capital. In 2020 between the 17th and 38th epidemiological week, over two percent of the 206,728 citizens were subjected to symptom analysis and RT-qPCR testing by the Federal University of Rio de Janeiro, with positive individuals being notified up to 48 h after swab collection. Geocodification and spatial cluster analysis were used to limit COVID-19 spreading in Macaé. Within the first semester after the outbreak of COVID-19 in Brazil, Macaé recorded 1.8% of fatalities associated with COVID-19 up to the 38th epidemiological week, which was at least five times lower than the state capital (10.6%). Overall, considering the successful experience of this joint effort of private and public engagement in Macaé, our data suggest that the development of a similar strategy countrywise could have contributed to a better control of the COVID-19 spread in Brazil. Quarantine decree by the local administration, comprehensive molecular testing coupled to scientific analysis of COVID-19 spreading, prevented the catastrophic consequences of the pandemic as seen in other populous cities within the state of Rio de Janeiro and elsewhere in Brazil.

## Introduction

Coronaviridae is a family of RNA viruses well-known to infect a large variety of mammalian and avian hosts^1^. The genus betacoronavirus is particularly important for public health, since at least five strains have already been reported to infect humans, leading to mild diseases, such as the common cold, pneumonia, as well as other severe respiratory illness. In the past two decades, previous severe respiratory disease infections by coronavirus, SARS-CoV and MERS-CoV, have provided evidence that these viruses could lead to pandemic outbreaks. The World Health Organization (WHO) reported a new strain of a coronavirus arising in China at the end of December 2019, with a strong pandemic potential^2^. In January 2020, SARS-CoV-2 was identified as the causative agent of Coronavirus Disease (COVID-19) and until August 30th of the same year, over 25 million people had already been infected with over 800,000 deaths being recorded globally^3^.

COVID-19 is characterized by dry cough and fever in about 60–70% of infected individuals, without major complications and medical intervention. However, 30–40% of those infected have evolved to severe respiratory disease, with 15–25% of individuals requiring long-term hospitalization with high mortality risk. Until September, 2020, Brazil had 4,455,386 confirmed cases of COVID-19 and 134,935 associated deaths, according to the Coronavirus Brazil Panel data (last updated on 9/17/2020 at 18:00 h; SVS/MS 2020). All over the country, testing has been generally limited to the ones with severe illnesses, and evidence suggests that COVID-19 related deaths in Brazil are greatly underestimated^4^.

The epidemiological situation in the state of Rio de Janeiro was of major concern because it encompasses the city of Rio de Janeiro. Rio de Janeiro is the second largest city in Brazil, with over 6.7 million people, 22% of whom live in densely inhabited slums under poor sanitary and household conditions^5^. The city of Rio de Janeiro was one of the first cities in Brazil to confirm COVID-19 cases. According to the Coronavirus Panel of Rio de Janeiro State Health Department, until 09/12/2020 at 18:44 h; health state secretary-RJ 2020, there had been 95.190 confirmed cases in the city of Rio de Janeiro, with 10.113 deaths. The case-fatality rate in the city was 10.6%, 3.5-fold higher than the country at that time (3.0%), suggesting that underreporting of cases in the country was substantial^6^.

Previous work indicated that the best strategy to overcome the disease is through large-scale testing, enabling a rapid diagnosis, and isolation of infected subjects, to halt viral transmission^7,8^. The absence of a nationally coordinated testing response during the first semester of 2020, urged for the action of public health actors, universities, local health authorities and hospitals, together with private corporations and banks, to increase test numbers^9^. In this context, the city of Macaé, located in the interior of the state of Rio de Janeiro capital and approximately 200 km from Rio de Janeiro city, adopted a comprehensive molecular (PCR) testing strategy followed by rapid isolation of positive individuals for COVID-19. This strategy was achieved by an alliance between the municipality government and donations from private sectors, which allowed a faster and cost-free molecular testing for the local population. In this article, data analyses of 4639 RT-qPCR tests, provided important insights of disease symptoms development, age and gender trends of positivity and deaths, as well as the neighborhood spatial distribution of COVID-19 in Macaé.

Overall, Macaé displayed the lowest lethality rate in the state of Rio de Janeiro during the analyzed period (April–September 2020), which, at least partially, reflected the prompt isolation of individuals with positive outcomes. However, this was only possible with an increase in the diagnostic rate and patient isolation effectiveness after April 12th. These data suggest that a similar nationally coordinated testing strategy between Brazilian public universities, research centers, government and private sectors could have contributed to decrease COVID-19 deaths at other municipalities around the country.

## Methods

### Inclusion criteria, nasopharyngeal swab collection, RNA extraction and RT-qPCR

From April 12th to September 12th, 2020 (17th–38th epidemiological weeks), a total of 4639 inhabitants displaying symptoms indicative of COVID-19 were medically examined at the municipal Coronavirus Screening Center for COVID-19 (CSC) or at one of the four hospitals involved in the study. At each place, healthcare professionals recorded the patient's vital signs, applied an admission questionnaire, which covered essential epidemiological data, including the presence-absence of 16 clinical symptoms, as well as relevant personal information (residential address and work activity). Study inclusion criteria included subjects with a clinical diagnosis of COVID-19, a Real-time reverse transcription polymerase (RT-qPCR) test for SARS-CoV-2 (positive or negative result), and the acceptance of a term of consent for the use of information. The study was approved by the Comitê de Ética em Pesquisa (Research Ethics Committee, Brazilian Ministry of Health: approval number 32868720.4.0000.5699). All research was performed in accordance with the relevant guidelines and regulations. Informed consent was obtained from all participants and/or their legal guardian(s). Study exclusion criteria included lack of willingness or ability to provide the informed consent or lack of an appropriate legal guardian or representative to provide the informed consent or other medical contraindication to donate nasopharyngeal sample. RT-qPCR tests were performed at the Institute NUPEM-UFRJ. For viral RNA extraction magnetic beads (Magmax Magnetic Kit—Thermofisher), were used following manufacturer’s instructions. Alternatively, this kit was substituted by the fast commercial extraction solution EasyExtract™, (Interprise^®^), as recently described^10,11^. RT-qPCR reactions for the identification of SARS-CoV-2 positive samples were performed using a TaqMan™ approach, as previously described in the Berlin^12^ or the CDC^13^ protocols. RT-qPCR tests were considered positive when two regions of SARS-CoV-2 genomes were amplified. Patients were considered uninfected when amplification was successful only for the human internal control (RNAse P). All RT-qPCR assays were performed on a StepOnePlus™ Real Time PCR System (Applied Biosystems).

### RT-qPCR positive case notification and symptom data analyses

From April 12th to September 12th, 2020 (a total of 22 epidemiological weeks), detailed medical information of 3495 individuals tested was recovered. 24 to 48 h after nasal swab collection, every individual with a positive RT-qPCR test received the notification from the Health Secretary Municipality in the Coronavirus Screening Center for COVID-19 (CSC). Proper orientation, either to remain isolated at home during the following 2 weeks, if mildly symptomatic or to attend the municipal hospitals, if necessary, was provided by the municipality.

The proportion of positive results were compared between gender among groups of individuals separated by age according to WHO, with the first age class being from 0 to 14 years old, and, then, from 15 to 80 years old, the age-class interval was every 5 years, totalizing 15 age classes. Additionally, comparisons were also made among age classes that encompass professional activities (between 15 and 74 years old) and among the days after the first symptom onset, using the Test of Equal or Given Proportions, which assumes equal proportions in two or more groups. The test of Equal or Given Proportions was also performed to compare the rate of positive outcomes between two temporal windows of 11 epidemiological weeks. The three possible types of cointegration (Type 1: no trend; Type 2: linear trend; Type 3: quadratic trend) between absolute mortality and positive results series of two earlier epidemiological weeks with the Engle-Granger Test were tested. The 2 weeks lag was chosen after checking the observation that 14 days (± 0.69 standard error) was the average time elapsed between RT-qPCR test and death of 75 fatal victims of COVID-19 in our cohort at Macaé.

Since the 16 symptoms included in the questionnaires presented a qualitative nature (1–0 or presence-absence of a given symptom), we employed a multivariate analysis technique to explore the similarity of symptoms on CSC positive results between men and women from the 15 age classes. Multivariate analysis have been widely used to explore the relationships of several characteristics measured on a number of individuals or communities, including microorganisms^14,15^. Herein, we dealt with a matrix of pairwise distances or dissimilarities between individuals based on their reported symptoms with the aim to ordinate them within a dissimilarity space limited to a low dimensional representation. Since multivariate analyses are generally sensitive to zero-inflated matrices, we excluded the three least reported symptoms (drowsiness, irritability and mental confusion), which were reported by less than 5% of the individuals. The Jaccard index was chosen to construct the distance-based matrix, since it is the most appropriate metric when variables are defined as 0–1. The distance-based matrix was then submitted to a non-metric dimensional scaling (NMDS) with the function *metaMDS* in the ‘vegan’ package^16^ in R 3.6.1, for visualization of the individuals on the two-dimensional space, according to their dissimilarity. Individuals presenting similar sets of symptoms are positioned close to one another, while those with dissimilar symptoms (e.g., not sharing any symptom) are positioned further apart. Compared to other ordination techniques, NMDS is recommended even when the relationship between dissimilarities and inter-object distances is nonlinear, as long as low stress values are obtained^17^. The stress value (from 0 to 1.0) measures how good the graphical representation is of the actual dissimilarities on the distance-based matrix. According to Quinn and Keough^17^, stress values greater than 0.3 indicate that the configuration is no better than arbitrary and therefore should not be interpreted.

Significant effects of gender and/or age class and their interaction on the distance-based matrix were investigated with a two-way permutational analysis of variance (PERMANOVA) performed with the function *adonis* in the 'permute' package^18^ in R 3.6.1. By expecting that the NMDS would be able to reveal structure on the most and less shared symptoms among individuals, we descriptively compare the scores between men and women among age classes along the first and second NMDS axes (NMDS1 and NMDS2, respectively) and related them to the prevalence of the symptoms.

### Comparison of viral cycle thresholds (Cts)

The ∆Ct approach was chosen to compare the approximate viral load of nasopharyngeal swabs, where Cts values of RNAse P (RP) endogenous control were subtracted from the arithmetic mean of N1 and N2 targets of SARS-CoV-2 (∆Ct = (CtN1 + CtN2)/2 – CtRP), for each positive patient^19,20^. The arithmetic mean of Ct values is usually employed when using multiple reference genes in relative analyses of gene expression studies^21^, and was used here to the targeted genes under the assumption that the expression of N1 and N2 viral genes are not differentially regulated between infected individuals. When compared to the presentation of crude Ct values, the ∆Ct approach has the advantage of providing a normalized Ct score, discounting for initial variations on swab collected biological material and also for the presence of possible polymerase inhibitors in the RT-qPCR reactions.

RT-qPCR positive patients were classified as mild or severe based on criteria previously defined by Liu et al.^19^, which include any of these conditions: respiratory distress (respiratory frequency ≥ 30 breaths/min), oxygen saturation at rest ≤ 93%, severe disease complications leading to hospitalization or death in consequence of the infection. We also attempted to classify these patients in early and late infected cases based on the day of appearance of the first COVID-19 symptoms, as reported in the admission questionnaires. The boundary up to and after the 5th day from manifestation of the first symptoms was chosen to establish early and late classes (respectively < 5 and > 5 days), as the proportions of mild and severe cases were comparatively balanced between these two intervals.

The ∆Ct of individuals were compared between mild and severe cases and between < 5 and > 5 days after the first symptom. Additionally, interactions among them were compared by analyses of variance (ANOVA), after checking for the residuals homoscedasticity with the function *leveneTest,* available in the package carData^22^.

### Geocodification, spatial distribution and spatial cluster analysis

The death notification forms from the Brazilian Ministry of Health's Notification and Surveillance System were extracted from the Municipal Health Department of Macaé. The address data (street address, number, neighborhood and zip code) were used for the geocoding process for either RT-qPCR positive results (cases) or deaths. To describe the spatial distribution of COVID-19 occurrences in the municipality, each case location was plotted together with the case density level curves, estimated using the Kernel smoothing method. The smoothed relative risk (SRR) was used to assess the spatial distribution of RT-qPCR positive results and COVID-19 deaths^23^. This analysis allowed the comparison of deaths caused by COVID-19 among neighborhoods. To estimate the SRR, the geocoded cases or deaths were grouped by neighborhood, and indirect standardization^35^ was used to compute the expected number of cases or deaths for each neighborhood. The SRR then follows as the ratio of the observed number of events (cases and deaths) over the expected number:

1 $$SRRi=Oi/Ei$$

where *Oi* represents the observed number of COVID-19 cases (deaths) in the area (neighborhood) *i*, and *Ei* represents the expected number of COVID-19 cases (deaths) for the area *i.*

To assess the spatial dependence of the distribution of COVID-19 cases (deaths), the Moran's I coefficient of autocorrelation was calculated to obtain the SRR. Autocorrelation statistics for aggregated data provide an estimate of the degree of spatial similarity observed among neighboring values of an attribute over a study area^24^. In general, if the SRR values in the dataset are clustered spatially (high values cluster near other high values; low values cluster near other low values), the autocorrelation method of Moran's Index will be positive. When high values repel other high values, and tend to be near low values, the Index will be negative.

The spatial analyses of the data and generation of the maps were performed using R 3.6.1. The Google Maps API was adopted from the R software package ggmap^25^ to geocoding, the tmap package^26^ was used to plot the weekly maps of cases or deaths. The smooth relative risks were estimated using the Dcluster package^27^ and the package spdep^28^ were used for the estimates and tests the Moran’s I coefficient.

## Results

### Macaé fatality rate was lower compared with other populous municipalities

**Figure 1:**
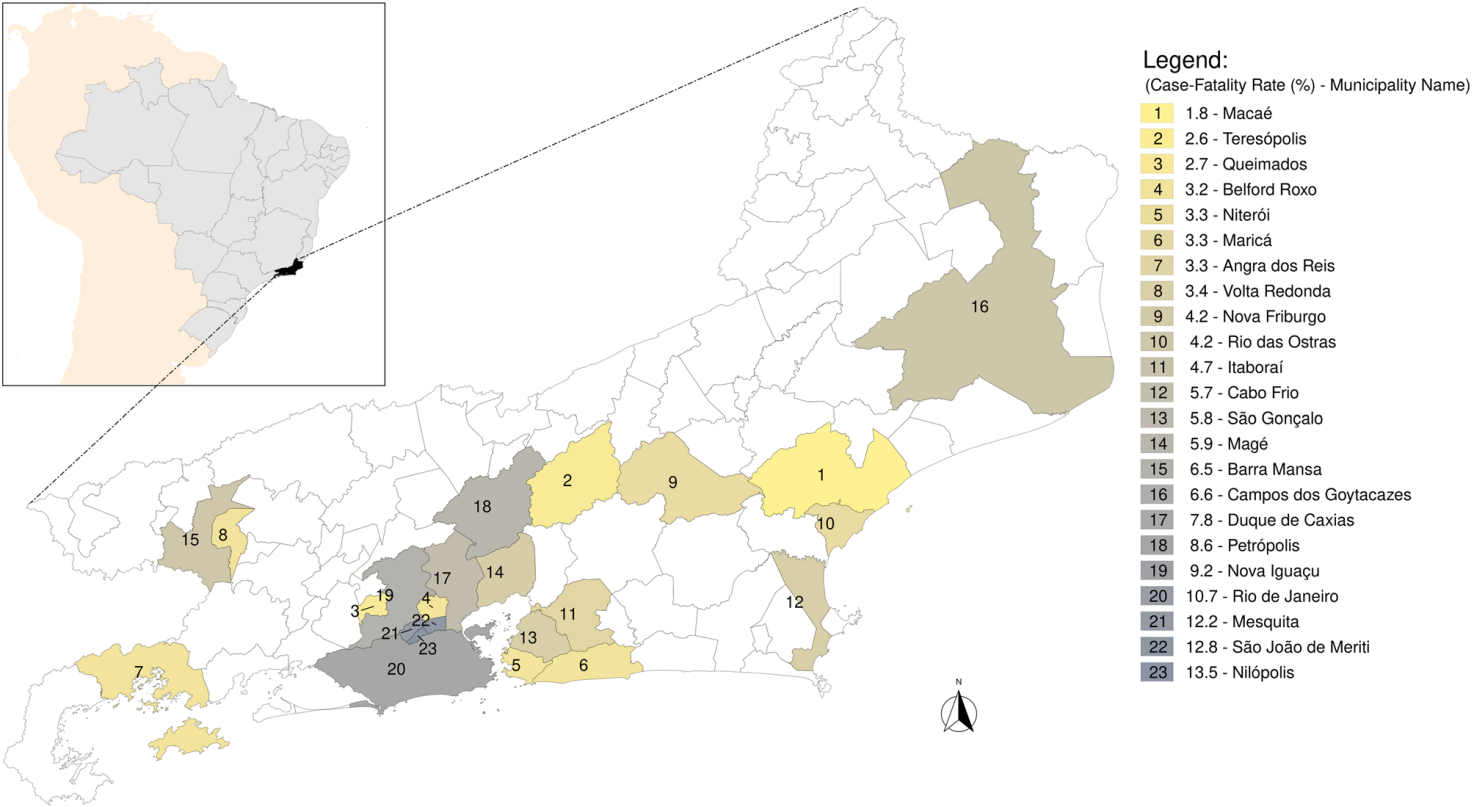
Macaé, the National Oil Capital, occupying the lowest fatality rate due to COVID-19 among the 23 most populous municipalities (> 125,000 inhabitants) of the state of Rio de Janeiro, Brazil. South America Background from rnaturalearth package^45^. Brazilian map, shape file from IBGE ftp://geoftp.ibge.gov.br/organizacao_do_territorio/malhas_territoriais/malhas_municipais/municipio_2019/Brasil/BR/br_unidades_da_federacao.zip. Rio de Janeiro map from IBGE, shape file . The figure was generated by the authors using the R 4.0.3 software^46^ and the packages rgdal^47^, ggspatial^48^ and tidyverse^49^ available at ftp://geoftp.ibge.gov.br/organizacao_do_territorio/malhas_territoriais/malhas_municipais/municipio_2019/Ufs/RJ/rj_municipios.zip.

Macaé is located in the State of Rio de Janeiro, southeast of Brazil (Fig. 1). Comparison of COVID-19 death rates showed that in 6 months of epidemic (up to the 38th epidemiological week), Macaé had the lowest mortality rate among the 23 most populous municipalities (> 125,000 inhabitants) in the state of Rio de Janeiro (Fig. 1).

### Men actively working contracted COVID-19 at higher rates than women

During the study period, 297 women and 286 men between 15 and 74 years tested positive for COVID-19. Comparison of both genders showed a higher rate of men engaging in working activity compared to women (Chi^2^ = 24.681; d.f. = 1; p < 0.001). A finer age scale interval showed an even clearer pattern, with men from the age of 35 onwards exerting a significantly higher working activity during quarantine than women (Age classes 15–34 years Chi^2^ = 1.150; d.f. = 1; p = 0.284; 35–54 years Chi^2^ = 25.40; d.f. = 1; p < 0.001; 55–74 years Chi^2^  =  8.036; d.f. = 1; p = 0.005) (Supplementary Fig. 2).

### Two distinct stages of COVID-19 spreading were identified in Macaé

**Figure 2:**
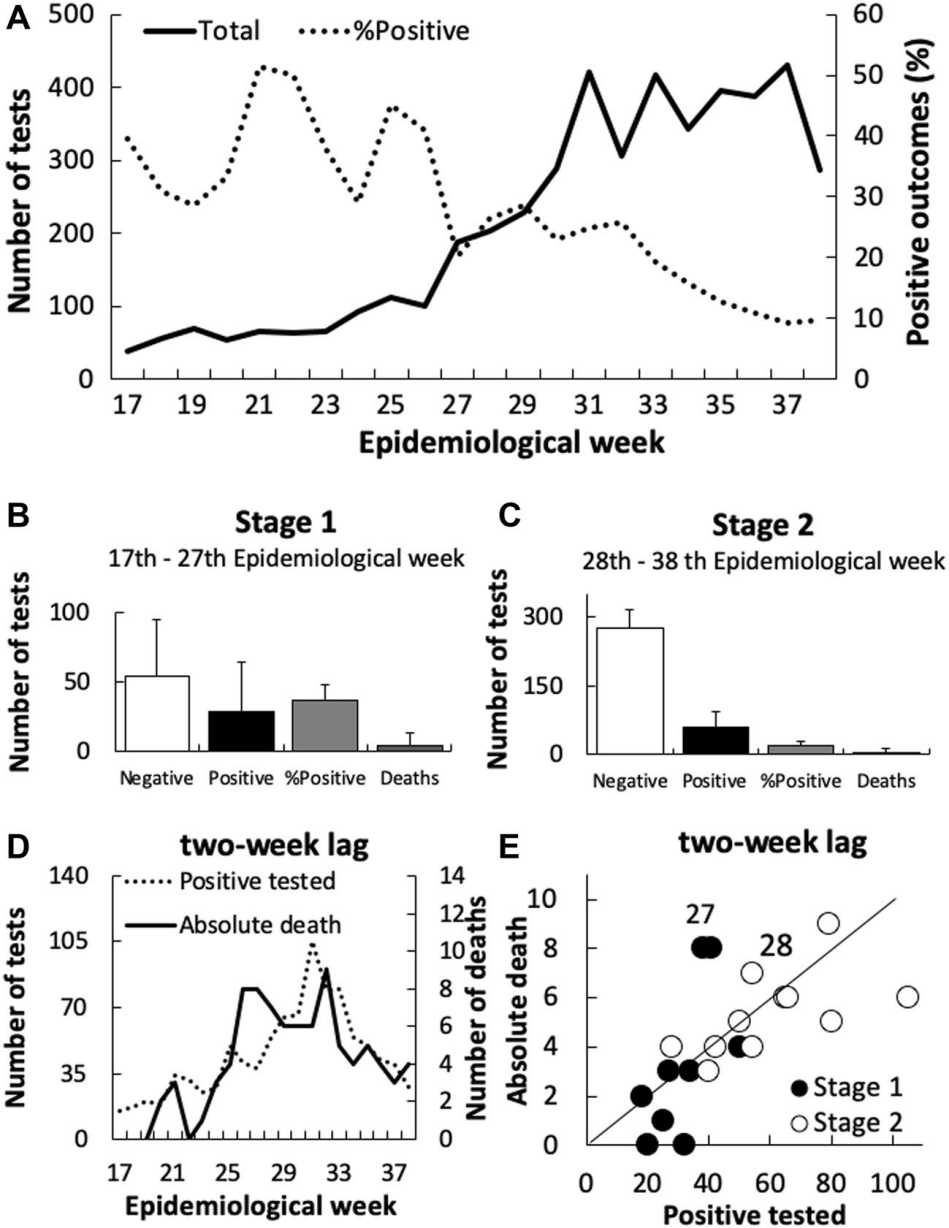
Relationship between the 4,611 COVID-19 RT-qPCR tests and positive result rates in Macaé, State of Rio de Janeiro, Brazil, between April 12th and September 12th, 2020. Note scale differences in (**B**) and (**C**). The diagonal in (**E**) represents a 1:10 ratio in absolute death and positive outcomes.

The sampling processing at the NUPEM-UFRJ laboratory increased from an average of 38 to 430 tests per week, until reaching a stable rate of 400 tests, from the 31th epidemiological week onwards (Fig. 2A). The ratio of positive RT-qPCR decreases throughout the epidemiological weeks, even though high fluctuations were recorded along the first 11 weeks (Fig. 2A). There were significant differences in the positive rates between two temporal windows, the first characterized by a low number of tests (Stage 1; Fig. 2B) and the other by a high number of tests (Stage 2; Fig. 2C) (Chi^2^ = 126.62; d.f. = 1; p < 0.001). A congruence between the number of positive tested and absolute deaths started to be observed only after the 32th epidemiological week (Fig. 2D) and this trend may explain the lack of cointegration between the number of positive results and the number of deaths for Types 1 (EG = − 2.07; p = 0.10), 2 (EG = 0.01; p = 0.10) and 3 (EH = 0.95; p = 0.10). On Stage 2, a constant ratio of 1:10 between fatality and positive outcomes was observed (Fig. 2E), even though both variables diminished with time.

### RT-qPCR tests and the occurrence of positive results

**Figure 3:**
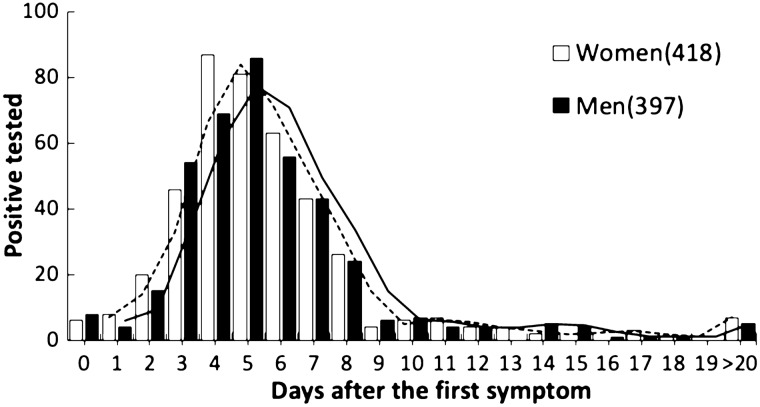
Distribution of the 815 positive tests for RT-qPCR COVID-19 by gender and days since the first symptom. This analysis is limited to patients from the municipal Coronavirus Screening Center for COVID-19 (CSC), Macaé, state of Rio de Janeiro, Brazil, between April 12th and September 12th, 2020. 155 cases were excluded from the analysis, since the day of the first symptom was not available.

Most positive RT-qPCR results were obtained from samples collected between the fourth and sixth day after the first symptoms (55% of the women and 53% of the men) and there was no significant difference between gender on the overall representativeness regarding the day of swab collection relative to first symptoms onset (Chi^2^ = 18.077; d.f. = 19; p = 0.517) (Fig. 3).

### Respiratory symptoms are associated with hospitalization

**Figure 4:**
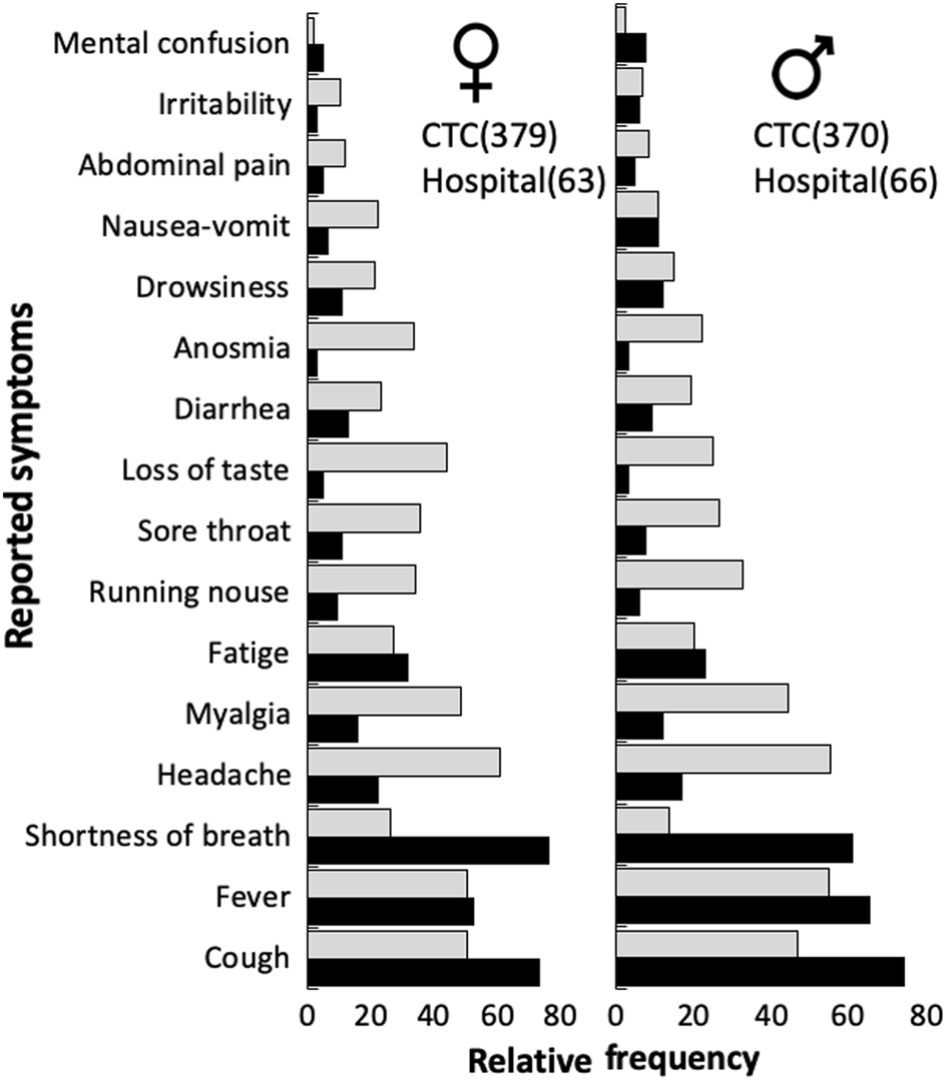
Prevalence of symptoms reported by 878 patients that tested positive for COVID-19 in CSC and the hospital settings of Macaé, state of Rio de Janeiro, Brazil, between April 12th and 12th September 2020. Number of positive tests are in brackets.

Among the 3751 tested for COVID-19 at the health center CSC and the hospital settings, 878 tested positive (51% from CSC and 49% from hospital). Symptoms related to respiratory disorders (i.e., cough and shortness of breath) were the most frequently reported by those requiring mostly intensive care in hospital settings, whereas varied and less frequent symptoms were more frequently reported by those with milder symptoms (Fig. 4). Among the latter, typical symptoms of influenza, such as headache, myalgia, runny nose and sore throat were similarly reported by women and men, whereas loss of taste, anosmia and nausea and vomit were more typically reported by women (Fig. 4; Supplementary Fig. 3). Nevertheless, irrespective of gender, individuals younger than 40 years old reported more frequently headache, loss of taste, anosmia and sore throat, while individuals older than 40 reported more frequently myalgia and fever (Supl. Figure 2). Independently of gender, deaths were concentrated in individuals older than 50 years (Supplementary Fig. 4).

### Type and frequency of symptoms vary depending on age and gender

**Figure 5:**
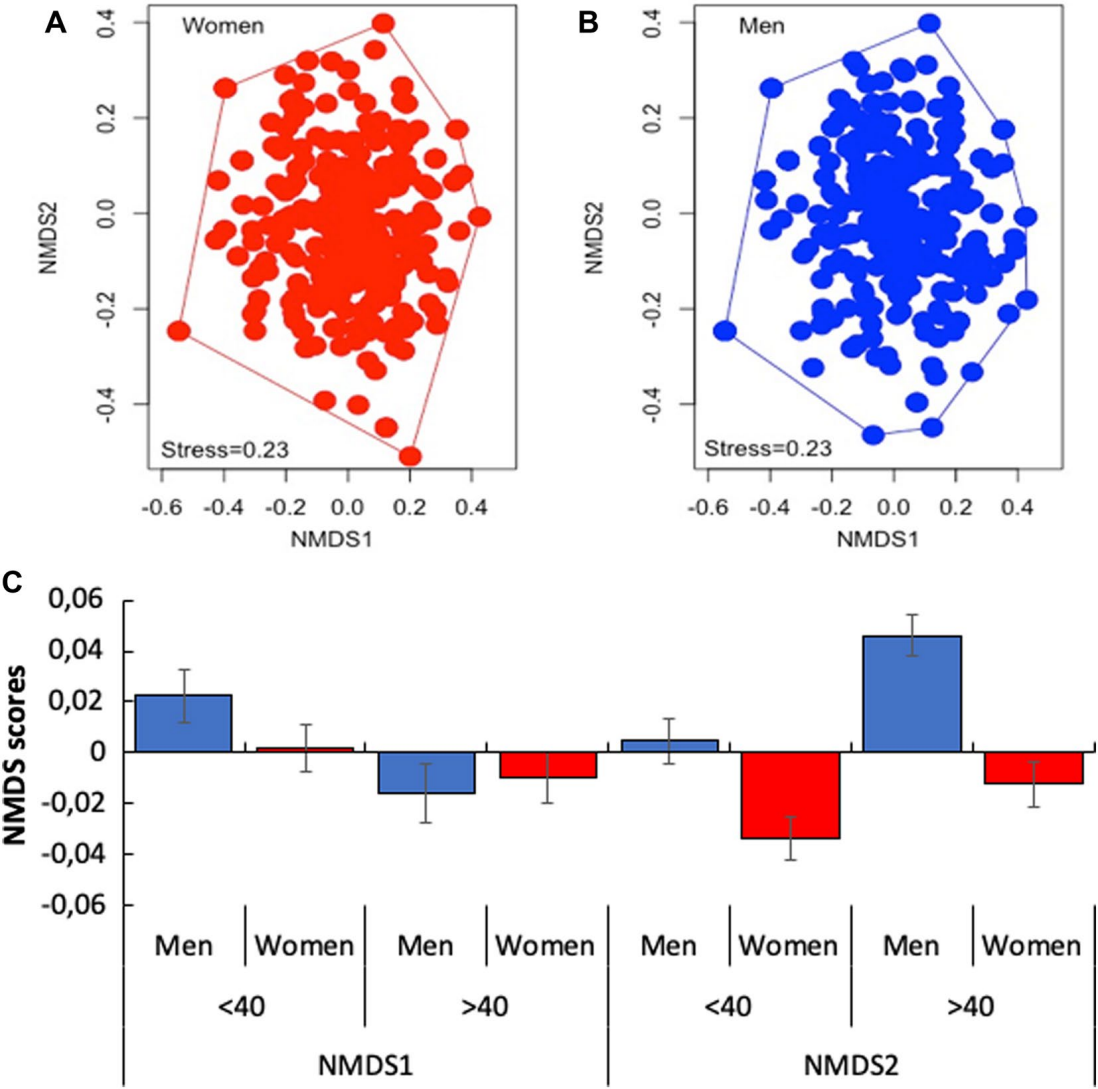
Ordination of women (**A**) and men (**B**) positively tested for COVID-19 in the municipal Coronavirus Screening Center for COVID-19 (CSC) on a multidimensional space formed by the two axes of the NMDS. Mean scores (± standard error) between gender and age class in C.

NMDS showed a widely spread ordination of the positive tested men and women, reinforcing a general pattern of heterogeneity of their symptoms (Fig. 5A,B). Since a stress value of 0.23 was observed in our dataset, the ordination was considered adequate^12^. The centroids of the ordinated individuals significantly differ between gender and age classes (Fig. 5C). PERMANOVA analysis detected significant differences within both effects, age (< 40 and > 40) and gender classes (Table 1; Supplementary Fig. 3).

**Table 1:** Permutational analysis of the effect of gender and age class on the matrix of the 13 symptoms reported by 707 patients that tested positive for COVID-19 RT-qPCR at the municipal Coronavirus Screening Center for COVID-19 patients (CSC) in Macaé, State of Rio de Janeiro, Brazil, between April 12th and September 12th, 2020.

	d.f.	Square sum	Mean square sum	Pseudo F	R^2^	P
Gender	1	1.598	1.598	7.129	0.010	0.001
Age class	14	5.002	0.357	1.594	0.031	0.001
Gender × age class	14	2.940	0.210	0.937	0.018	0.616
Error	676	151.548	0.224		0.941	
Total	705	161.088			1.000	

Detailed analysis showed that those significant differences among individuals are associated with the milder symptoms, such as loss of taste, anosmia and nausea-vomit, that prevailed among women (Supplementary Fig. 3). In addition, there was also an effect of age on the type and frequency of the reported symptoms. Interestingly, irrespective of gender, individuals younger than 40 years old reported more frequently headache, loss of taste, anosmia and sore throat, while individuals older than 40 reported more frequently myalgia and fever (Table 1; Supplementary Fig. 3). These trends are reinforced by the comparison of the scores of positive tested individuals along the first two NMDS axes (Fig. 5C). Age has a stronger effect along NMDS1 (younger individuals with positive and older with negative scores, respectively) and gender along NMDS2, where men presented positive scores, whereas women presented negative scores (Fig. 5C).

### Higher ∆Ct values from nasopharyngeal RT-qPCR swab tests are not associated with stronger disease severity

**Figure 6:**
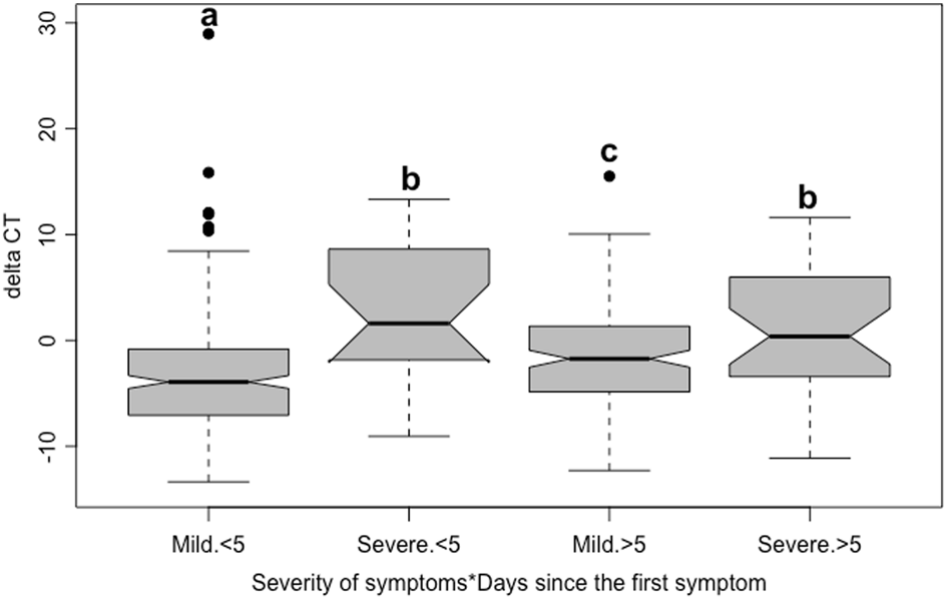
∆Ct of SARS-CoV-2 positive patients with mild and severe symptoms of COVID-19. Patient RT-qPCR results were separated in four different groups based on days after the first symptoms and severity of the disease. Mild < 5 are ∆Ct values from 257 patients with mild COVID-19 symptoms tested until 5 days from the first symptoms (89% of them from the municipal Coronavirus Screening Center for COVID-19 patients-CTC), Severe < 5 are 20 patients hospitalized (80% of them) early after infection, or non-hospitalized patients with severity-related symptoms or still COVID-19 associated fatalities. The same classification was adopted for the patients with more than 5 days of symptoms:158 Mild > 5 (85% of them from the CTC) and 32 Severe > 5 (78% of them from hospitals). Significant differences among groups are represented by different letters above boxplots.

To understand if the differences of patients with mild and severe previously described COVID-19 symptoms (see Fig. 4) can also be associated with changes in the viral load from nasopharyngeal swabs, a RT-qPCR comparative analysis was performed using the ∆Ct values as a response variable (see^29^ and references therein). Lower ∆Ct values directly correspond to higher viral load in nasopharyngeal swabs. The results showed that the viral load is clearly reduced with time after the appearance of the first symptoms, but this is perceptible only for mild COVID-19 patients (Fig. 6). In contrast, patients presenting severe symptoms showed a lower viral load (higher ∆Ct values), independent of the day of the RT-qPCR test was performed (< 5 or > 5) (Table 2).

**Table 2:** Effect of the severity (mild or severe) of the disease (based on the records of the vital rates) and days after the first symptoms (< 5 or > 5 days) based on a two-way analysis of variance (ANOVA).

	d.f.	Square sum	Mean square sum	F	P
Severity	1	927	926.9	32.635	2.02e−08
Threshold 5 days	1	193	193	6.794	0.009
Severity * threshold 5 days	1	133	133.4	4.698	0.031
Error	453	12,866	28.4		

RT-qPCR ∆Ct values obtained from 457 individuals that tested positive for COVID-19 at the municipal Coronavirus Screening Center for COVID-19 (CSC) and the hospital settings of Macaé, State of Rio de Janeiro, Brazil, between April 12th and September 12th, 2020.

### Highly populated neighborhoods contain COVID-19 RT-qPCR positive hotspots and deaths

**Figure 7:**
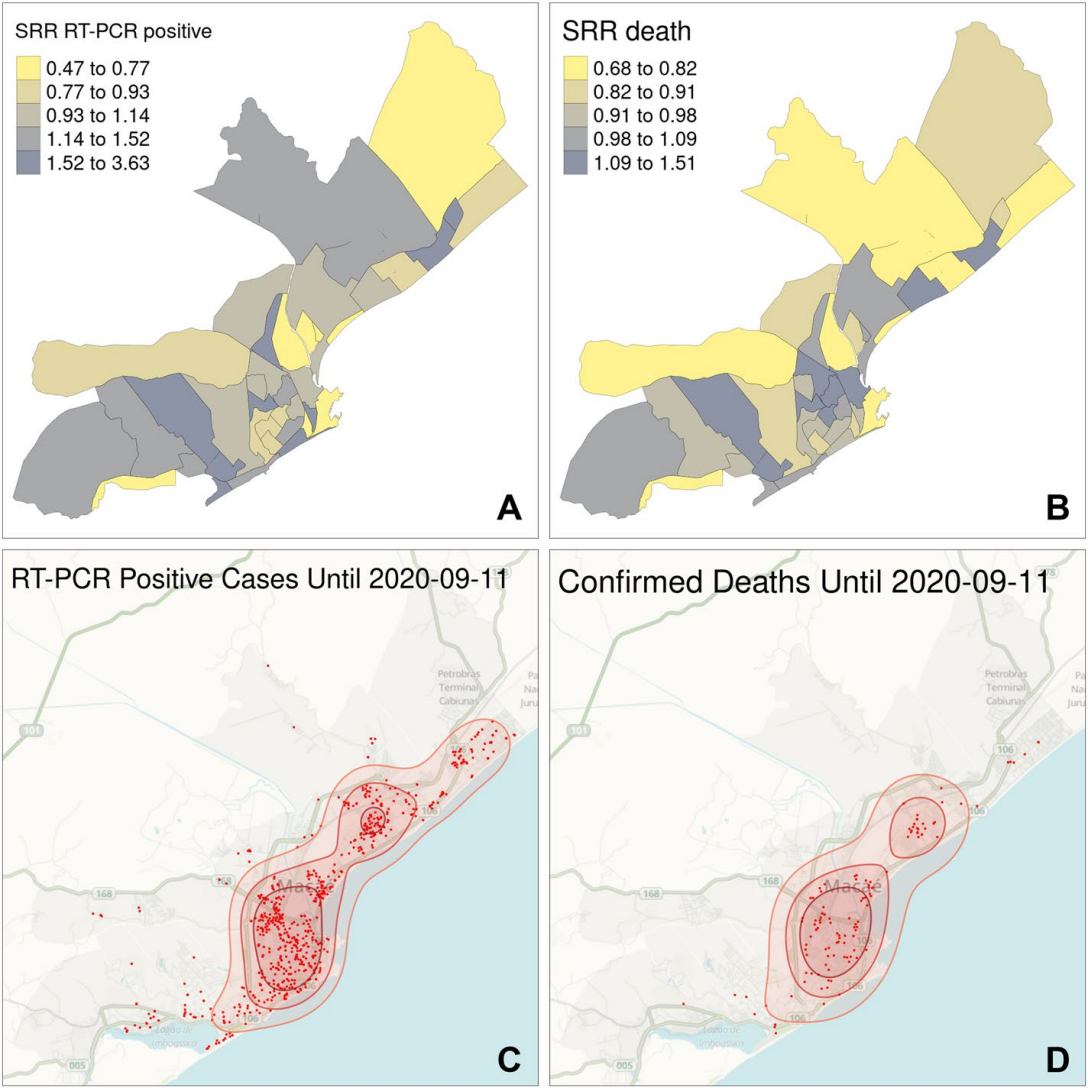
Spatial distribution of accumulated RT-qPCR positive cases and deaths in Macaé, Rio de Janeiro. (**A**,**B**) Map representation of the different neighborhoods of Macáe, RJ and SSR representation of RT-qPCR positivity (**A**) and deaths (**B**). (**C**,**D**) The exact locations of each occurrence (case and death) and the density level curves with 25, 50 and 75% of the estimated cases using the Kernel smoothing method. Background from mapmisc package^50^. Macaé map from GeoMacaé, shape file available at http://www.macae.rj.gov.br/midia/conteudo/arquivos/1452711889.zip. The figure was generated using the R 4.0.3 software^46^ and the packages rgdal^47^, tmap^26^, adehabitatHR^51^ and tidyverse^49^.

The smoothed relative risk (SRR) analysis allowed the comparison of RT-qPCR positivity and deaths rates among neighborhoods. Observation of SRR quintiles of positive RT-qPCR cases (Fig. 7A) and of urban deaths (Fig. 7B) showed a widespread distribution of SRR values across the city. The Moran Index statistic results were − 0.035 (p-value = 0.5264) for SRR positive RT-qPCR and − 0.019 (p-value = 0.3555) for SRR deaths, confirming that COVID-19 infections and deaths were evenly distributed along the city and not spatially concentrated. The weekly comparative analysis of RT-qPCR positivity and deaths shows that at the 17th epidemiological week, positive RT-qPCR cases were already spread along the city (Sup. Figures 4, 5). Interestingly, there is a large correspondence between positive RT-qPCRs and deaths (Fig. 7C,D), thus, regions with high RT-qPCR positivity were also the ones corresponding to high death occurrences. Comparison of these data with population density (Supplementary Fig. 7) also highlights that positive RT-qPCRs and deaths were concentrated in neighborhoods in which the greatest number of inhabitants are found.

## Discussion

Rio de Janeiro is one of the most populous Brazilian states and even though it has a diversified economy, the state relies mainly on the extraction of natural resources, such as oil and gas^30^. Located 200 km north of the state capital, Macaé has attracted dozens of oil and hundreds of outsourced companies in the last 50 years, which renders the municipality the title of the National Oil Capital^31^. Since the beginning of March 2020, when Macaé recorded the first COVID-19 cases, the municipal governance began publishing a series of decrees (available at^32^) that included the closure of schools and temples, restriction of all non-essential activities, rigorous control of the arrivals by installation of sanitary barriers on all major municipality entries and the creation of the municipal Coronavirus Screening Center for COVID-19 patients (CSC), in the city center. Additionally, an alliance between the municipal governance, the Institute of Biodiversity and Sustainability of the Federal University of Rio de Janeiro in Macaé (NUPEM-UFRJ), several oil industry related companies and hospitals, allowed the implementation of a new laboratory that was urgently adapted for such a sanitary emergency, with the aim to set up the gold standard technique for SARS-Cov2 identification, the RT-qPCR. Consequently, from the 17th epidemiological week onwards (April 12th, 2020), samples from nasopharyngeal swabs from CSC and four associated hospitals were collected and delivered to a recently created laboratory at NUPEM-UFRJ for RT-qPCR analysis.

Positive individuals were notified 24 to 48 h after collection, enabling for a fast and proper treatment of symptomatic patients in hospitals and the recommendation of isolation of mildly symptomatic or asymptomatic individuals in their houses. The strengthened of the selection criteria of patients for RT-qPCR that arrived more severely ill at the CSC on the previous epidemiological weeks and the effectiveness of the governance policies against the spread of the virus may explain the more positive rates between the 17th to 27th epidemiological weeks (Stage 1). In the present study we use correlation data, thus, it was not possible to unveil if the large number of RT-qPCR tests in Macaé, during early stages of the disease, in fact, led to a reduction in ascertainment bias when compared to the rest of the state, since there is a possibility that the mobility restrictions by the municipality also played a role. However, mobility restrictions were performed all over the state (and throughout the country), and thus, restriction measures alone cannot explain the lowest death rates in Macaé, compared to the rest of the state or the country^6,9^.

**Figure 8:**
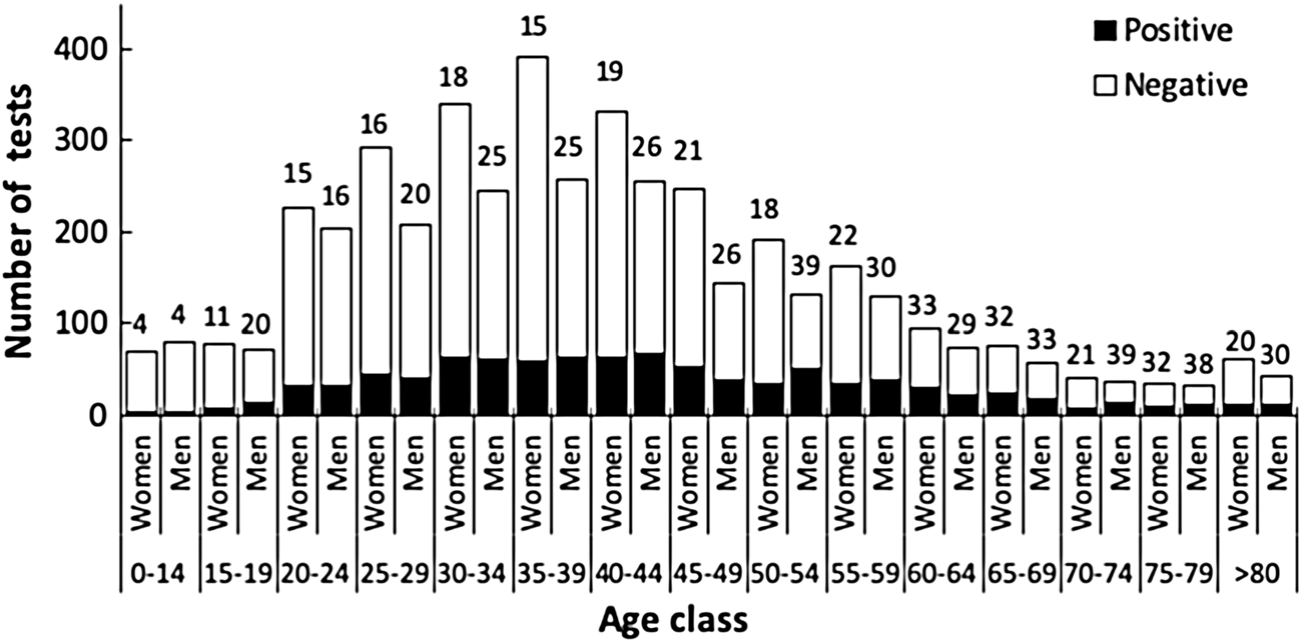
Number and percentage of positive individuals among the 4,639 tests for COVID-19 RT-qPCR in Macaé, State of Rio de Janeiro, Brazil, according gender (Men = 1986; Women = 2653), between April 12th and September 12th, 2020. Numbers above columns refer to the percentage of positive results within each gender and age class. See Table 3 to identify age classes with significant differences between gender (in bold).

In the present study, it was observed a higher percentage of positive COVID-19 men at working ages (between 25 and 54) than women in Macaé (Fig. 8). Essential activities of offshore companies and transport are still generally men-based worldwide, and these sectors did not stop during the quarantine in Macaé. Research has shown that women are highly under-represented in most extractive industries^33^. The U.S. Department of Labor defines a male-dominated sector as one where women constitute less than a quarter of the total workforce^33^. On the other hand, public services, educational system and street commerce, which usually employs genders more equally, were strongly restrained by the municipal decrees. Some of those sectors (i.e., non-essential commerce) were re-opening their traditional activities at the beginning of the second half of 2020, whereas by October 18th 2020, presential activities in schools, universities and public departments had not yet restarted. Taken together, these results suggest that men actively working in Macaé were more exposed to infection and contracted COVID-19 at higher rates than women.

**Table 3:** Distribution of positive results between gender overall and within age class, among the 4611 COVID-19 RT-qPCR tests performed in Macaé, state of Rio de Janeiro, Brazil, between April 12th and September 12th, 2020.

Age class (years)	Total tested (N)		Total positive outcome (N)		Positive ratio		Equality in proportions		
	Men	Women	Men	Women	Men	Women	Chi^2^	d.f.	P
Overall	1971	2640	489	486	0.248	0.184	27.345	1	< 0.001
0–14	80	69	3	3	0.038	0.043	< 0.001	1	1.000
15–19	71	79	14	9	0.197	0.114	1.407	1	0.236
20–24	205	226	32	33	0.156	0.146	0.025	1	0.875
25–29	208	292	41	46	0.197	0.158	1.063	1	0.303
30–34	245	341	61	63	0.249	0.185	3.151	1	0.076
35–39	257	392	63	59	0.245	0.151	8.496	1	0.004
40–44	255	332	67	63	0.263	0.190	4.043	1	0.044
45–49	145	248	38	53	0.262	0.214	0.946	1	0.331
50–54	132	191	51	34	0.386	0.178	16.417	1	< 0.001
55–59	129	162	39	35	0.302	0.216	2.382	1	0.123
60–64	75	94	22	31	0.293	0.330	0.116	1	0.733
65–69	58	77	19	25	0.328	0.325	< 0.001	1	1.000
70–74	36	42	14	9	0.389	0.214	2.065	1	0.151
75–79	32	34	12	11	0.375	0.324	0.032	1	0.857
> 80	43	61	13	12	0.302	0.197	1.016	1	0.313

The fact that most positive RT-qPCR results were from samples collected between the fourth and sixth day after the first symptoms reinforces that the ideal window for doing the RT-qPCR is between 3 and 8 days after symptom onset, regardless of the gender tested. However, men and women differ on the type and the frequency of the reported symptoms, although symptoms are also dependent on age. COVID-19 symptoms have been described over 6 months by now^34^ and our data support previous observations that symptoms related to respiratory disorders (i.e. cough and shortness of breath) were most frequently reported by those requiring most intensive care in the hospital settings (Fig. 4). As previously observed^35^, absolute deaths increased within age classes, independent of gender in Macaé, Rio de Janeiro, Brazil, between April 12th and September 12th 2020. This age associated increase of COVID-19 mortality has been previously reported^35,36^, highlighting that age is one of the most important death risk factors of COVID-19. Altogether, our data provide a framework for clinical doctors to assess the most frequent symptoms which might lead to hospitalization.

The association between ∆Ct values and COVID-19 severity can also be discussed from our data. Briefly, there was no association between viral load, evaluated by ∆Ct values, and disease severity (Fig. 6). A study with a limited number of patients in Hong Kong was also unable to observe an association between viral load in posterior oropharyngeal or saliva samples and disease severity^37^. Interestingly, Argyropoulos et al.^38^ have reported a similar finding when comparing viral loads between hospitalized and non-hospitalized New York patients. These authors argued that higher viral loads observed in patients presenting mild symptoms may reflect the elapsed time from infection onset, as viral loads usually peak during the pre-symptomatic stage or shortly after the manifestation of first symptoms of COVID-19 and then show a slow decline during the following 2-weeks^39^. Then, while many patients recover from the disease in this 2-week period, a small proportion of them will suffer from progressive health deterioration, even with lower viral load using nasopharyngeal swabs. Thus, most patients identified as severe cases may have been infected and replicating SARS-CoV-2 several days before presenting the first symptoms. Together with the reports from^37,38^ our observations corroborate previous studies showing that long lasting COVID-19 syndrome is not necessarily correlated with higher viral loads, but rather with lower ones^39^. The representative sample number presented herein and the more robust statistical tests than most previous studies reinforces this conclusion. This is of special concern for health authorities, since individuals in earlier stages of the disease are more often asymptomatic or mildly symptomatic and, thus, may spread the disease more easily.

Another noteworthy finding from the current study comprised the COVID-19 positivity distribution throughout Macaé city (Fig. 7, Supplementary Figs. 5–7). Highly populated areas from the city containing banks, supermarkets and pharmacies concentrate both RT-qPCR positive and death cases, suggesting that these essential trade activities might foster disease spreading along the city. Importantly, population density and SSR RT-qPCR positivity were directly correlated with spatial death occurrence all over the city over the whole analyzed period (17th and 38th epidemiologic week), thus, authorities should focus on containing the spread of the disease mainly at these specific locations. Previous analysis at two states of Brazil, Ceará and São Paulo, also provided evidence that metropolitan highly populated areas showed the greatest number of COVID-19 cases^40,41^. Spread of the virus can be highly associated with the mobility of people by means of transportation, such as planes and buses^41^. Importantly, one of the hotspots of Macaé COVID-19 RT-qPCR data overlaps with the municipal bus station, providing further evidence that people´s mobility and population concentration are essential factors for disease spreading. Other modeling approaches using Susceptible-Infected-Removed (SIR) or SEIAQR (susceptible-exposed-infected-asymptomatic-quarantined-recovered) were used to analyse COVID-19 spread in Japan^42,43^. These studies estimated the effective reproduction number (Re), the transmission parameters, and the need to reduce the time spent in crowded locations to less than four hours. Independently of the modelling approach applied, RT-qPCR tests at early stages of infections were also proposed to be essential to reduce COVID-19 transmission^42,43^.

COVID-19 stable transmission in Brazil has been estimated to be established during early-mid March 2020 (11th to 12th epidemiological weeks) and differences in viral introductions were observed depending on the state. For instance, while in Ceará, Northeastern Brazil, genome sequences were grouped in a single clade, the Amazon state presented several international and national introductions during the pandemic^44^. In this case, Macaé might represent an important hotspot for introduction of new SARS-Cov-2 lineages in Brazil from other countries, since oil and gas workers arrive daily in the city. Thus, COVID-19 control in Macaé and other surrounding cities might be an additional challenge if new viral lineages are introduced from abroad, as new lineages are constantly being identified^44^.

To sum up, a well-designed strategy of testing and isolation of positive individuals was successful to mitigate the effects of COVID-19 in an important oil production city of Brazil, until September, 2020. Statistical analysis showed that respiratory symptoms are associated with a worse disease prognosis. Furthermore, geocoded spatial analysis indicated that highly populated areas display the largest number of RT-qPCR positive cases and deaths. Altogether, the multidisciplinary approach established here, coupling effective RT-qPCR tests, early individual notification and analysis of disease spreading, could have contributed to a better control of COVID-19 in Brazil if the methodology had been applied country wise.

## Supplementary Information

Supplementary Information.
