# Supplementary material for: Molecular testing and analysis of disease spreading during the emergence of COVID-19 in Macaé, the Brazilian National Capital of Oil
Source: Sci Rep. 2021 Oct 11;11:20121. doi: 10.1038/s41598-021-99475-7 (PMC8505656; doi:10.1038/s41598-021-99475-7)
Supplement: Supplementary file 1 — Supplementary Information. [file 41598_2021_99475_MOESM1_ESM.docx]

Molecular testing and analysis of disease spreading during the emergence of COVID-19 in Macaé, the Brazilian National Capital of Oil

Authors:

Natália Martins Feitosa^1*^, Bruno da Costa Rodrigues^1*^, Ana Cristina Petry^1*^, Keity Jaqueline Chagas Vilela Nocchi^1^, Rodrigo de Moraes Brindeiro^2^, Carla Zilberberg^1^, Cintia Monteiro-de-Barros^1^, Flavia Borges Mury^1^, Jackson de Souza Menezes^1^, José Luciano Nepomuceno-Silva^1^, Manuela Leal da Silva^1^, Marcio José de Medeiros^1^, Raquel de Souza Gestinari^1^, Alessandra da Silva de Alvarenga^1^, Allan Pierre Bonetti Pozzobon^1^, Carina Azevedo Oliveira Silva^1^, Daniele das Graças dos Santos^1^, Diego Henrique Silvestre^1^, Graziele Fonseca de Sousa^1^, Janimayri Forastieri de Almeida^1^, Jhenifer Nascimento da Silva^1^, Layza Mendes Brandão^1^, Leandro de Oliveira Drummond^1^, Lupis Ribeiro Gomes Neto^1^, Raphael de Mello Carpes^1^, Renata Coutinho dos Santos^1^, Taynan Motta Portal^1^, Amilcar Tanuri^2#^, Rodrigo Nunes-da-Fonseca^1#^

*shared first authors

*^#^ shared corresponding authors

**Supplementary figures:**


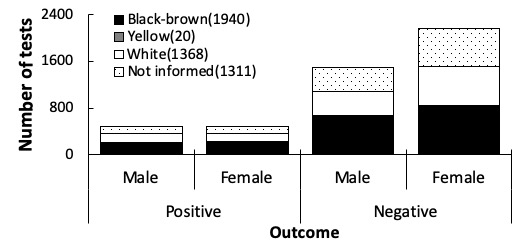


**Sup. Figure 1: Rates of the outcomes of the 4,639 COVID-19 RT-qPCR tests in Macaé, Rio de Janeiro, Brazil, according race/color (Positives = 978; Negatives = 3,661), between 13th April and 12th September 2020.** Number of tests in brackets. The 4,639 tests cover a relatively similar proportion gender for positive outcomes and it was women biased for negative outcomes. Ca. 30% of the individuals tested did not inform race/color, whereas the proportion of black and caucasians was similar.


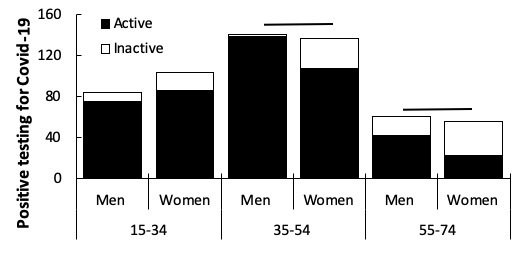


**Sup. Figure 2: Proportion among the 583 positive testing for COVID-19 RT-qPCR in Macaé, Rio de Janeiro, Brazil, between 13th April and 12th September 2020.** Only individuals confirming laboral activities during the quarantine (Men = 286; Women = 297) were considered for the analysis. The bars identify significant age classes with significant differences in proportion between gender.

**
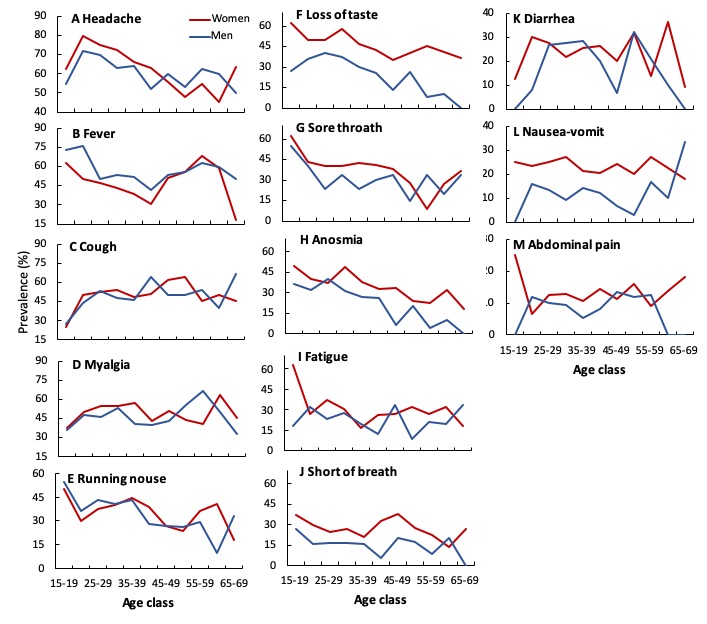
**

**Sup. Figure 3: Prevalence of the thirteen most reported symptoms among age classes of the 380 women and 370 men testing positive for COVID-19 at the municipal health center of Macaé, Rio de Janeiro, Brazil, between April 12th and September 12, 2020.** Only age classes with more than 15 individuals are presented (omitted 0-14, 70-74, 75-79, >80).


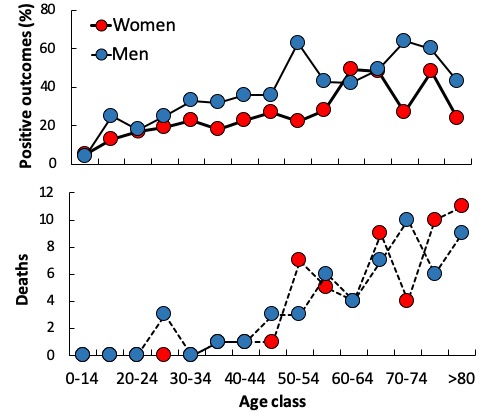


**Sup. Figure 4: Relationship between relative positive outcomes and absolute deaths within age classes among 3,487 COVID-19 RT-qPCR tests (Men = 1,510; Women = 1,977), in Macaé, Rio de Janeiro, Brazil.** Data from April 12th and September 12th 2020. Irrespective of gender, positive testing and mortality increased with age.


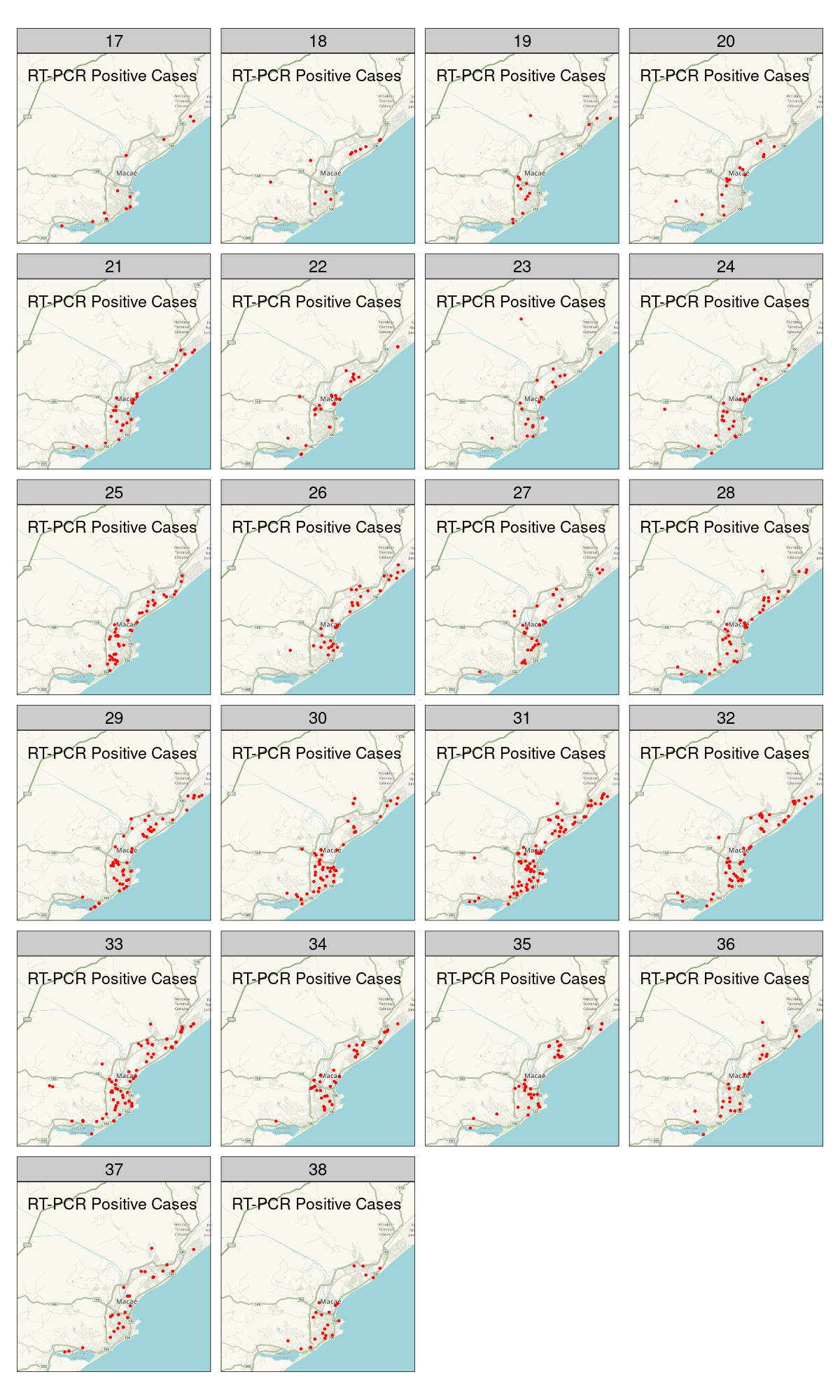


**Sup. Figure 5: Spatial distribution of positive RT-PCR week by week (17^th^ until 38^th^ epidemiological week) at Macaé city.** Background from mapmisc package [1]. Macaé map from GeoMacaé, shape file available at http://www.macae.rj.gov.br/midia/conteudo/arquivos/1452711889.zip. The figure was generated at R 4.0.3 software [2] using the packages rgdal [3], tmap [4], and tidyverse [5].


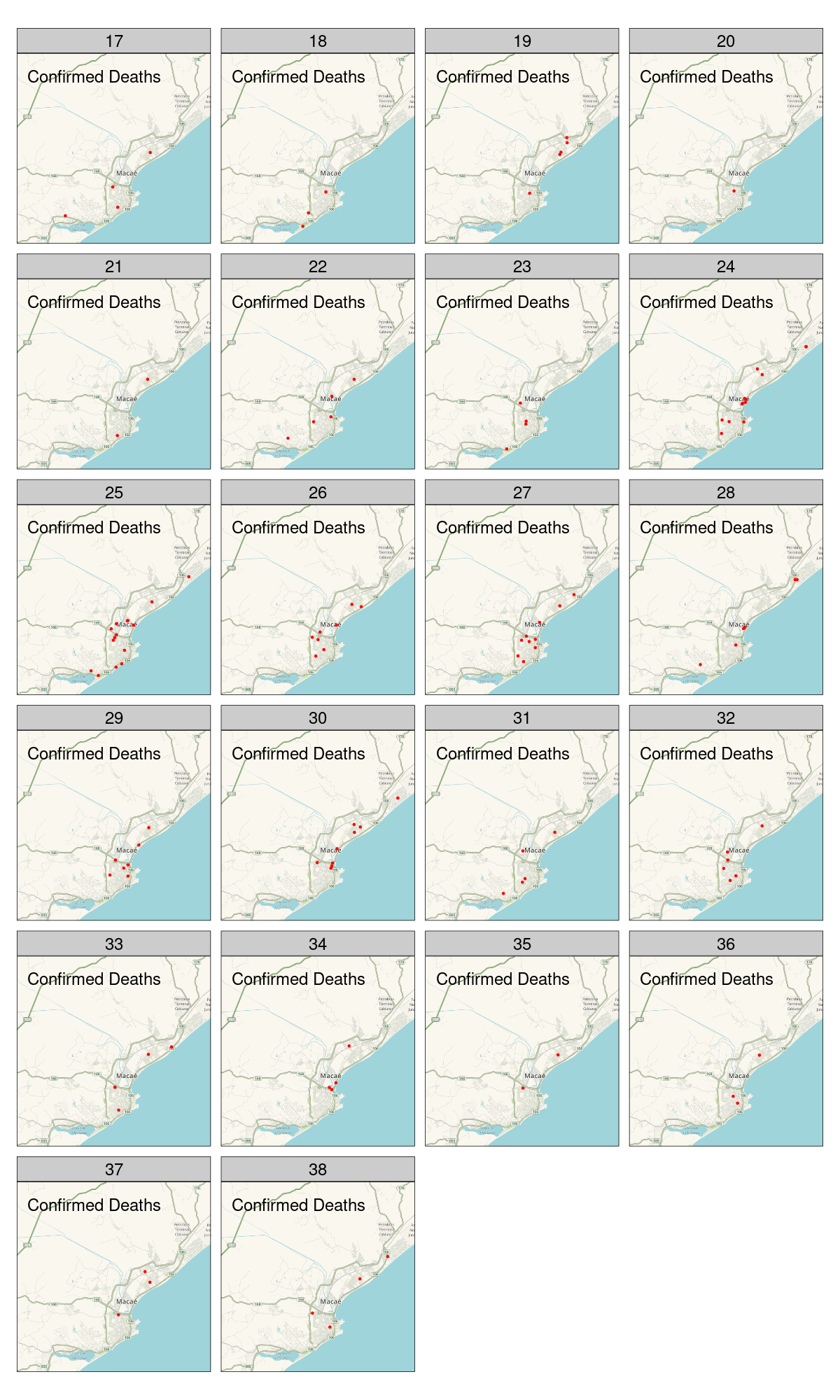


**Sup. Figure 6: Spatial distribution of deaths week by week (17^th^ until 38^th^ epidemiological week) at Macaé city.** Background from mapmisc package [1]. Macaé map from GeoMacaé, shape file available at http://www.macae.rj.gov.br/midia/conteudo/arquivos/1452711889.zip. The figure was generated at R 4.0.3 software [2] using the packages rgdal [3], tmap [4], and tidyverse [5].


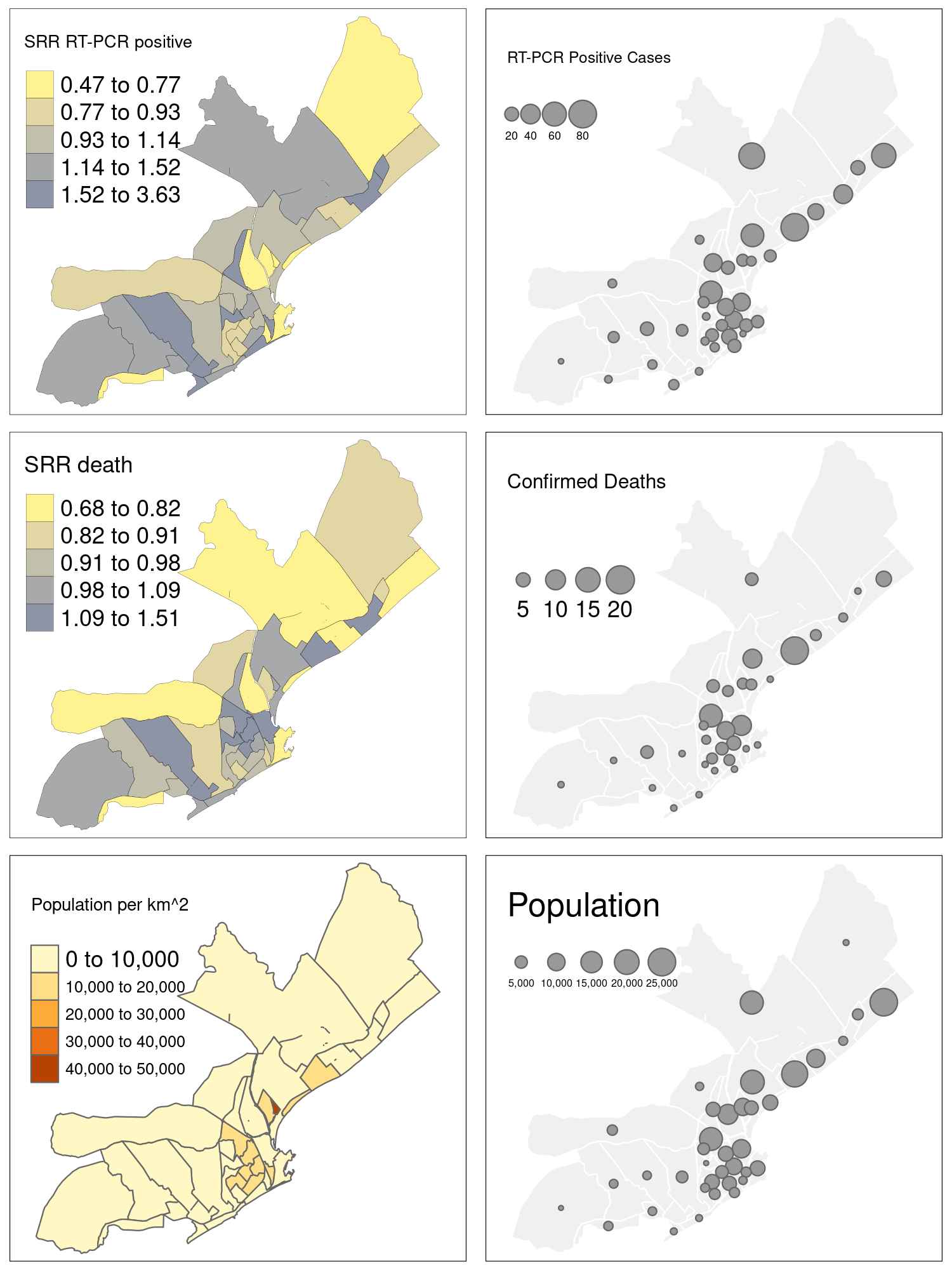


**Sup. Figure 7: Comparison of SRR values of RT-qPCR positive cases, deaths and population density of different Macaé neighborhoods.** In general, the highly populated areas also correspond to high numbers of RT-qPCR positive cases and deaths. Macaé map from GeoMacaé, shape file available at <http://www.macae.rj.gov.br/midia/conteudo/arquivos/1452711889.zip>. The figure was generated at R 4.0.3 software [2] using the packages rgdal [3] and tmap [4]

References:

1. P.E. B. Maps, coordinate reference systems and visualising geographic data with mapmisc. . R-journal. 2016;8(1): 64-91.

2. Team RC. R: A language and environment for statistical computing. R Foundation

for Statistical Computing, Vienna, Austria. . 2021.

3. Bivand R, Keitt T, Rowlingson B. rgdal: Bindings for the 'Geospatial' Data Abstraction Library. R package version 1.5-25. . 2021.

4. Tennekes M. tmap: Thematic Maps in R. Journal of Statistical Software. 2018;84(6):1–39. doi: 10.18637/jss.v084.i06.

5. Wickham H. Welcome to the tidyverse. . Journal of Open Source Software. 2019;4(43).
